# Characterization and expression analysis of genes encoding ubiquitin conjugating domain-containing enzymes in *Carica papaya*

**DOI:** 10.1371/journal.pone.0171357

**Published:** 2017-02-23

**Authors:** Dengwei Jue, Xuelian Sang, Bo Shu, Liqin Liu, Yicheng Wang, Zhiwei Jia, Yu Zou, Shengyou Shi

**Affiliations:** 1 Key Laboratory of Tropical Fruit Biology (Ministry of Agriculture), South Subtropical Crops Research Institute, Chinese Academy of Tropical Agricultural Sciences, Zhanjiang, China; 2 Rice Research Institute, Anhui Academy of Agricultural Sciences, Hefei, China; Universidade de Lisboa Instituto Superior de Agronomia, PORTUGAL

## Abstract

**Background:**

Ripening affects the quality and nutritional contents of fleshy fruits and is a crucial process of fruit development. Although several studies have suggested that ubiquitin-conjugating enzyme (E2s or UBC enzymes) are involved in the regulation of fruit ripening, little is known about the function of E2s in papaya (*Carica papaya*).

**Methodology/Principal findings:**

In the present study, we searched the papaya genome and identified 34 putative UBC genes, which were clustered into 17 phylogenetic subgroups. We also analyzed the nucleotide sequences of the papaya UBC (*CpUBC*) genes and found that both exon-intron junctions and sequence motifs were highly conserved among the phylogenetic subgroups. Using real-time PCR analysis, we also found that all the CpUBC genes were expressed in roots, stems, leaves, male and female flowers, and mature fruit, although the expression of some of the genes was increased or decreased in one or several specific organs. We also found that the expression of 13 and two CpUBC genes were incresesd or decreased during one and two ripening stages, respectively. Expression analyses indicates possible E2s playing a more significant role in fruit ripening for further studies.

**Conclusions:**

To the best of our knowledge, this is the first reported genome-wide analysis of the papaya UBC gene family, and the results will facilitate further investigation of the roles of UBC genes in fruit ripening and will aide in the functional validation of UBC genes in papaya.

## Introduction

Papaya (*Carica papaya*) is an economically important fruit crop that is widely grown in tropical and sub-tropical regions, and its fleshy fruits play an important role in the human food supply, as well as in human nutrition and health [1, 2]. Gaining in popularity among tropical fruits worldwide, papaya is now ranked fourth in total tropical fruit production after bananas, oranges), and mango [3]. Fruit ripening is an important and complex process, and, therefore, the regulatory mechanisms of fruit ripening have been intensively studied [4]. Fruit with different ripening mechanisms can be divided into two groups: climacteric fruit (e.g., bananas, tomatoes, pears, mangos, and papayas), in which ripening is accompanied by a peak in respiration and a concomitant burst of ethylene, and non-climacteric fruit (e.g., pineapple, carambola, borojo, and watermelon), in which respiration exhibits no dramatic change and ethylene production remains at a very low level [5].

Ethylene, in particular, has been extensively studied, owing to its crucial role in the ripening of climacteric fruits [6–9], and the biosynthesis, perception, and signal transduction of ethylene, as well as downstream gene regulation, are well known [10]. Tomato (*Solanum lycopersicum*) has long served as an excellent model for studies of fleshy fruit development and ripening [11, 12], and to date, many of the underlying genes have been cloned, thus providing insight into the regulation of ripening. The ripening-associated genes that have been reported in tomato include: *rin*, which encodes a MADS-box transcription factor [13]; *Nr*, which encodes an ethylene receptor [14]; *Cnr* (*Colorless nonripening*), which encodes an SBP-box transcription factor [15]; *Gr* (*Green ripe*), which encodes a still poorly defined component of the ethylene transduction pathway [6]; *SINAC4*, a new tomato NAC transcription factor that positively regulates fruit ripening and carotenoid accumulation [16]; *APETALA2a* (*AP2a*), a transcription factor that regulates fruit ripening *via* regulation of ethylene biosynthesis and signaling [17]; *LeNCED1*, which initiates abscisic acid (ABA) biosynthesis at the onset of fruit ripening and might act as an original inducer in tomato [18]; and the tomato genes *SHATTERPROOF*, *TAGL1* [19], *TDR4/FUL1*, and *MBP7/FUL2* [20], which are also involved in regulating fruit ripening. However, in contrast to tomato, there have been only a few reports regarding fruit ripening in *C*. *papaya*.

Ubiquitination is an essential cellular process in all eukaryotes, from unicellular yeast to humans, and the ubiquitination-proteasomal pathway has been implicated in diverse aspects of eukaryotic cellular regulation, owing to its ability to degrade intracellular proteins [21, 22]. The process of protein ubiquitination is mediated through the action of three enzymes, which are known as ubiquitin-activating enzymes (E1s), ubiquitin-conjugating enzymes (E2s), and ubiquitin ligases (E3s) [23]. Ubiquitin-conjugating enzymes (E2s) function at an intermediate step in the protein ubiquitination pathway. The E2 family has expanded during evolution, and more ancestral eukaryotes possess fewer E2 enzymes (e.g., ≤20 in algae) than more derived ones (e.g., >40 in certain plants and animals) [24]. For example, 13 UBC domain proteins have been identified in *Saccharomyces cerevisiae* [25], 12, 18, and 19 E2 enzymes are encoded in the algae *Ostreococcus tauri*, *Micromonas sp*. *RCC299*, and *Chlamydomonas reinhardtii*, respectively (data not shown), 20 in *Caenorhabditis elegans* [26], 37 in both humans [24] and *Arabidopsis thaliana* [27], 48, 52, 74 and 75 UBC domain proteins in rice, tomato, banana and maize [28–31].

Previous studies have suggested that ubiquitination plays an important role in plant growth, development, and tolerance against various abiotic stresses, and several of the UBC genes in *A*. *thaliana* have been implicated specifically [32, 33]. For example, the *A*. *thaliana* ubiquitin-conjugating gene *AtUBC13* has been implicated in epidermal cell differentiation and iron deficiency responses [34, 35], and AtUBC32 is an endoplasmic reticulum-associated degradation (ERAD) component that functions in brassinosteroid-mediated salt stress tolerance [36], whereas AtUBC21 (AtPEX4) is specialized for ubiquitination in peroxisome maintenance [37]. In rice, 14 *OsUBCs* were differentially expressed under drought and salt stress conditions [38], and in *Zea mays*, 48 and 16 *ZmUBC* genes were significantly upregulated under salt and drought stress conditions, respectively [30].

Several studies suggest that E2s also participate in the fruit ripening process. In tomato, for example, *SlUBC32*, which is upregulated during tomato fruit ripening and downregulated in the rin mutant, plays an important role in the regulation of fruit ripening [31]; and in banana, 32 *MaUBC* genes are reportedly up- and downregulated during different ripening stages [29]. However, functional studies of E2s involved in fruit ripening are limited and have not been conducted in papaya. Therefore, in the present study, we performed a genome-wide analysis of E2 domains, analyses of CpUBC gene structures and phylogenetic relationships, and expression profiling of CpUBC genes in various *C*. *papaya* plant tissues and at different stages of fruit ripening. To the best of our knowledge, this is the first reported genome-wide analysis of the papaya UBC gene family, and the results will facilitate further investigation of the roles of the UBC genes in fruit ripening and will aide in the functional validation of UBC genes in papaya.

## Materials and Methods

### 2.1. Plant material and treatments

Papaya (*C*. *papaya* cv. ‘sun up’) plants were obtained from the South Subtropical Crops Research Institute of the Chinese Academy of Tropical Agricultural Science (Zhan-jiang, Guangdong Province, China), and root, stem, leaf, male flower, female flower, and fruit (mature green) organs were collected separately. For the fruit ripening experiment, we also collected fruit pulp from four development stages: immature green (IG), mature green (MG), breaker (Br), and mature fruit (MF). The experiments were performed in triplicate, and all samples were immediately frozen in liquid nitrogen and stored at −80°C for expression analysis.

### 2.2. Sequence retrieval and gene family member identification

To identify potential members of the papaya UBC protein family, published *Arabidopsis* and yeast (*S*. *cerevisiae*) UBC protein sequences were retrieved and used as queries in BLASTP searches against the *C*. *papaya* Genomics Database (CpGDB; http://www.plantgdb.org/CpGDB/) and PLAZA 3.0 (http://bioinformatics.psb.ugent.be/plaza/versions/plaza/) [39]. Amino acid sequences of all the UBC-encoding genes of *Arabidopsis*, *S*. *cerevisiae*, and *S*. *lycopersicum* were downloaded from the *Arabidopsis* Information Resource (http://www.arabidopsis.org/), *Saccharomyces* Genome Database (http://www.yeastgenome.org/), and Sol Genomics Network Tomato Database (http://solgenomics.net) websites, respectively. All putative papaya UBC protein sequences were then subject to domain analysis using Simple Modular Architecture Research Tool (SMART; http://smart.emblheidelberg.de/) [40], and information regarding the CpUBC genes, including location, open reading frame (ORF), amino acid (AA) length and intron number, were obtained from PLAZA 3.0 [39]. The molecular weight (MW) and theoretical isoelectric point (PI) of the candidate CpUBC proteins were investigated using ExPASy online tools (http://expasy.org/tools/) [41]. Finally, the structure of each gene was visualized using the Gene Structure Display Server (GSDS) (http://gsds.cbi.pku.edu.cn/) [42].

### 2.3. Sequence alignment and phylogenetic analysis

To evaluate the evolutionary relationship of the CpUBC proteins, full-length amino acid sequences of 34 CpUBC proteins, 15 UBC proteins from *S*. *cerevisiae*, 48 UBC proteins from *Arabidopsis thaliana*, and 52 UBC proteins from *S*. *lycopersicum* were subject to multiple sequence alignment, using MUSCLE [43], with default parameters, and manual optimization. We excluded ambiguously aligned sequences, in order to produce an alignment of 34 amino acid characters for subsequent phylogenetic analyses. An unrooted phylogenetic tree was constructed using the neighbor-joining (NJ) method in MEGA (Version 6.0; http://www.megasoftware.net/), and the significance of nodes was assessed using a bootstrap test with 1,000 iterations [44]. Representations of the calculated trees were constructed using TreeView (Version 1.6.6; http://taxonomy.zoology.gla.ac.uk/rod/treeview).

### 2.4. RNA isolation and expression analysis

Total RNA was extracted for cDNA synthesis using the Super fast new plants of RNA extraction kit (Huayueyang Bio Co., Ltd, Beijing, China), following the manufacturer's instructions. Reverse transcription reactions were performed using the PrimeScript RT reagent kit with gDNA Eraser (Takara Bio, Inc., Kusatsu, Japan), according to the supplier’s manual. Real-time PCR was performed using a Bio-Rad real-time thermal cycling system (LightCycler 480; Bio-Rad Laboratories, Inc., Hercules, CA, USA) and SYBR-green to assess the expression levels of 34 candidate CpUBC genes. The gene-specific primers were designed according to the CpUBC gene sequences using Primer 5 software and checked using Blast (S1 Table) In addition, the papaya actin 1 gene was used as an internal control for normalization. Each reaction consisted of 10 μL 2× SYBR Premix Ex Taq II (Takara Bio), 40 ng cDNA, and 250 nM of each primer, in a final volume of 20 μL. The following PCR program was used: 94°C for 10 min, followed by 40 cycles of 94°C for 10 s, 58–63°C for 20 s, and 72°C for 30 s. The relative mRNA levels of the genes were measured using the cycle threshold (Ct) 2^**(-ΔCt)**^ method. The analysis included cDNA from the three biological samples for each tissue, and all the reactions were run in triplicate. In the comparative expression analysis of *CpUBCs*, genes that were up- or downregulated by at least two-fold were considered differentially expressed.

## Results

### Identification of UBC genes in papaya

In the present study, we used the published *Arabidopsis* and yeast (*S*. *cerevisiae*) UBC protein sequences as queries in BLASTP searches against the CpGDB and PLAZA 3.0[39], a total of 39 putative CpUBC genes were identified. After scanning of the 39 sequences for the UBC domain by motif scan using SMART search, we found only 34 sequences contain the UBC domain. According to their chromosome locations (Table 1), the 34 CpUBC genes were designated *CpUBC1* to *CpUBC34*. In these 34 CpUBCs, two were identified as Related to Ubiqutin (RUB) conjugating enzymes (CpUBC18 and CpUBC29), and one was identified as a SUMO-conjugating enzyme (CpUBC11). Six other UBC proteins (CpUBC2/14/16/19/24/25) lacked the Cys active site, identifying them as ubiquitin-conjugating enzyme variants (UEVs), which are not active by themselves, thus leaving 25 potential ubiquitin E2s. To better understand the papaya E2s, we constructed a recombinant E2 protein library of all 34 CpUBCs. The predicted proteins ranged from 98 (CpUBC15) to 668 amino acids (CpUBC20) in length, with corresponding molecular masses of 10.74 kDa and 74.03 kDa, respectively, and predicted isoelectric points of between 4.29 (CpUBC33) and 9.64 (CpUBC27).

**Table 1:** The information of *CpUBC* gene family.

Gene name	Gene locus	Chromosome Location	ORF(bp)	Size(aa)	PI	MW(KDa)	Intron
***CpUBC1***	CP00001G03780	supercontig_1: 4880545–4883588	504	167	5.04	18.72	4
***CpUBC2***	CP00002G01990	supercontig_2: 2835597–2840351	474	158	5.11	17.96	4
***CpUBC3***	CP00002G02590	supercontig_2: 3448167–3450541	447	148	7.72	16.48	3
***CpUBC4***	CP00003G00420	supercontig_3: 241676–245597	447	148	7.72	16.59	3
***CpUBC5***	CP00004G01190	supercontig_4: 2377968–2380915	585	194	4.69	21.28	4
***CpUBC6***	CP00006G01800	supercontig_6: 1485889–1488676	492	163	8.35	18.6	5
***CpUBC7***	CP00006G02820	supercontig_6: 2177997–2178675	447	148	8.44	16.55	3
***CpUBC8***	CP00009G00470	supercontig_9: 221437–222339	903	300	9.22	33.92	0
***CpUBC9***	CP00012G00790	supercontig_12: 606495–607675	148	447	7.72	16.46	3
***CpUBC10***	CP00017G01980	supercontig_17: 2605422–2607916	480	159	5.81	18.15	4
***CpUBC11***	CP00021G01620	supercontig_21: 1651878–1654594	483	160	8.42	18.03	4
***CpUBC12***	CP00023G00940	supercontig_23: 1591010–1594089	1593	530	6.33	58.62	5
***CpUBC13***	CP00027G00240	supercontig_27: 207634–210077	1170	389	4.99	43.83	4
***CpUBC14***	CP00033G01420	supercontig_33:1629710–1630930	1221	406	6.14	42.23	0
***CpUBC15***	CP00037G01340	supercontig_37:1138171–1138798	297	98	5.18	10.74	2
***CpUBC16***	CP00052G00760	supercontig_52: 720742–721809	936	311	5.41	35.21	1
***CpUBC17***	CP00053G00580	supercontig_53: 377001–380351	576	191	5.55	21.32	5
***CpUBC18***	CP00062G01080	supercontig_62: 722413–724323	612	203	8.53	23.25	5
***CpUBC19***	CP00073G00060	supercontig_73: 180777–182000	1224	407	6.68	45.56	0
***CpUBC20***	CP00080G00590	supercontig_80: 464531–468738	2007	668	4.66	74.03	7
***CpUBC21***	CP00084G00040	supercontig_84: 64510–66540	546	181	5.21	20.02	5
***CpUBC22***	CP00092G00550	supercontig_92: 491412–493626	780	259	8.69	28.16	4
***CpUBC23***	CP00097G00940	supercontig_97: 653771–662278	462	153	6.74	17.22	7
***CpUBC24***	CP00104G00590	supercontig_104: 426794–432223	441	146	6.2	16.62	3
***CpUBC25***	CP00119G00620	supercontig_119: 447043–449670	387	128	8.62	13.73	2
***CpUBC26***	CP00122G00250	supercontig_122: 616176–622862	447	148	7.72	16.55	3
***CpUBC27***	CP00124G00190	supercontig_124: 451236–454332	360	119	9.64	14.04	3
***CpUBC28***	CP00129G00500	supercontig_129: 420388–423075	465	154	4.91	17.22	5
***CpUBC29***	CP00158G00160	supercontig_158: 234845–236579	552	183	7.69	20.99	4
***CpUBC30***	CP00161G00220	supercontig_161: 289660–296412	459	152	5.37	17.32	4
***CpUBC31***	CP00183G00210	supercontig_183: 206758–210983	459	152	5.31	17.30	4
***CpUBC32***	CP00190G00050	supercontig_190: 25441–28352	426	141	5.58	16.14	4
***CpUBC33***	CP00214G00120	supercontig_214: 202216–221651	552	183	4.29	20.78	5
***CpUBC34***	CP01258G00030	supercontig_1258: 3731–7220	555	184	4.41	21.09	5

### Structure and phylogenetic analysis of CpUBC genes

**Fig 1:**
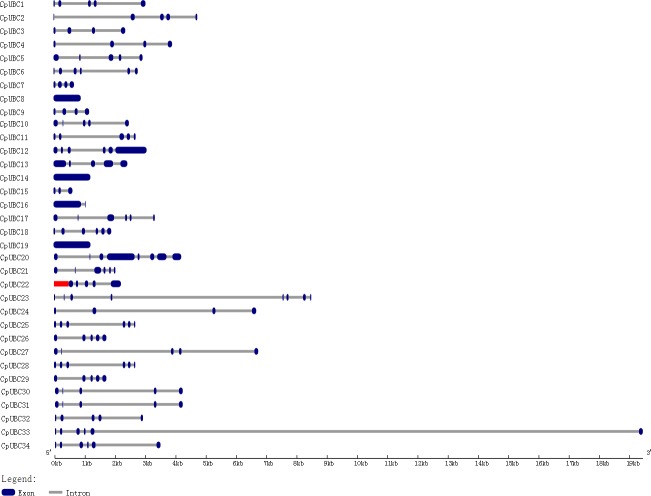
Structure of CpUBC genes. Untranslated 5′ and 3′ regions, exons, and introns are indicated by red, blue, and gray, respectively. The scale bar represents 1000 bp.

Using the GSDS website, we found that the number of introns in the 34 CpUBC genes ranged from zero (*CpUBC8*, *CpUBC14* and *CpUBC19*) to seven (*CpUBC20* and *CpUBC23*), with most of CpUBC genes containing three to five introns (n = 76.5%) (Table 1 and Fig 1). In addition, most CpUBC genes within the same subfamilies shared the same exon/intron structure. For example, in the UBC4/5 subfamily, *CpUBC3*, *CpUBC4*, *CpUBC7*, *CpUBC9*, and *CpUBC26* contained three introns, whereas in the UBC11 subfamily, *CpUBC17* and *CpUBC21* harbored five introns.

**Fig 2:**
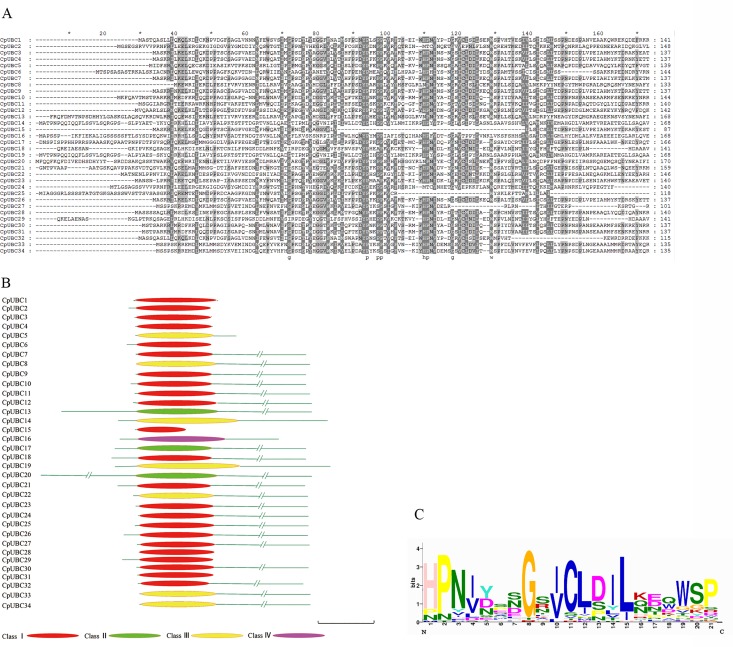
The multiple sequence alignment, structures and the architecture of conserved protein motifs of CpUBC proteins. (A) Multiple sequence alignment of CpUBC domains. Identical amino acids, conserved amino acids, and blocks of similar amino acid residues are shaded in black, charcoal gray, and gray, respectively. (B) Structures of the CpUBC proteins. The name of each corresponding protein is shown on the left. The position of the UBC domain is indicated in the figure. The length and order of the domains represent their actual location within each protein. The scale bar represents 100 amino acids. The different colors indicate the four E2 subtypes of UBC domains. (C) Conserved CpUBC domains. Logos of the protein alignment of the UBC domain is shown. The x-axis represents the conserved sequences, and the conservation of each residue is indicated by the height of each letter. The y-axis is a scale of the relative entropy, which reflects the conservation rate of each amino acid.

Multiple sequence alignment of the predicted amino acid sequences revealed that the tryptophan (W) located at the C-terminal side of the active cysteine was conserved in most of the CpUBC genes (Fig 2A). The consensus active site motif “HPN” was found at six amino acids from the N-terminal site of the active cysteine, and the strongly conserved PxxPP motif was found at seven amino acids from the N-terminal side of the HPN motif (Fig 2A). In addition, the predicted amino acid sequences of the UBC domains of 34 CpUBC genes were analyzed using the MEME Suite website (http://meme-suite.org/index.html). The results indicated that the highly conserved sequence of the UBC domain is HPNINSNGSICLDILKEQWSP (Fig 2C).

According to the UBC domain and the N- or C-terminal structure, E2 proteins are divided into four types. Class I E2s contain only the catalytic domain; Class II E2s contain N-terminal extensions; Class III E2s contain C-terminal extensions; and Class IV E2s have both N- and C-extensions [45, 46]. In the present study, we found that 23 of the CpUBCs belonged to Class I, and three, seven, and one belonged to Class II, Class III, and Class IV, respectively (Fig 2B).

**Fig 3:**
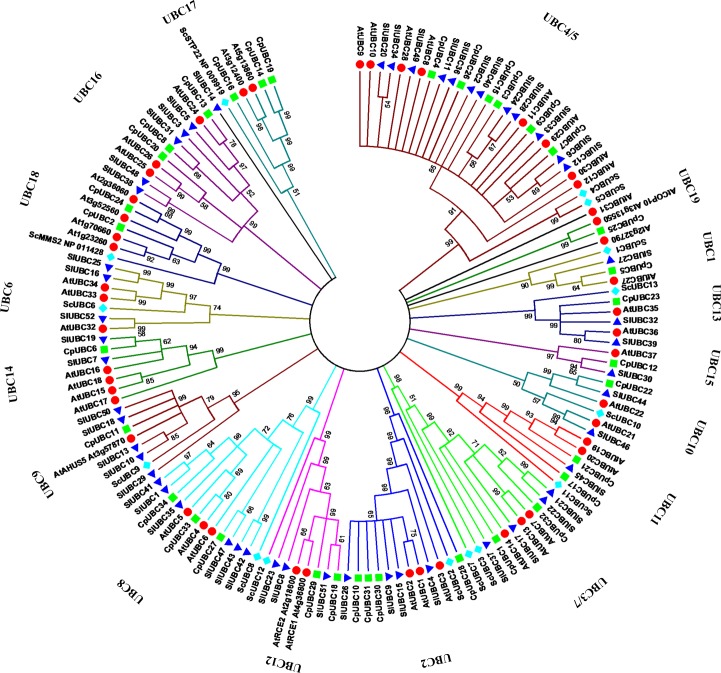
Phylogenetic tree of ubiquitin-conjugating (UBC) domains from *Carica papaya*, *Arabidopsis*, *Solanum lycopersicum*, and *Saccharomyces cerevisiae*. The neighbor-joining tree includes 34 UBC proteins from papaya (squares), 15 from *S*. *cerevisiae* (rhombi), 48 from *A*. *thaliana* (circles), and 52 from *S*. *lycopersicum* (triangles). The different colors indicate different CpUBC subgroups.

Furthermore, phylogenetic analysis revealed that the UBC proteins could be divided into 14 E2 groups and three independent UEV groups (UBC17, UBC18, and UBC19, with UBC9 and UBC12 functioning in the SUMO and RUB1 conjugation pathways, based on >50% bootstrap support (Fig 3). Most of the CpUBC groups were named according to the identities of the *S*. *cerevisiae* proteins or proteins contained within the group (UBC1, UBC2, UBC3, UBC4/5, UBC6, UBC3/7, UBC8, UBC9, UBC10, UBC11, UBC12, UBC13, UBC17, and UBC18); however, there were no yeast proteins in groups UBC14, UBC15, UBC16, and UBC19, which indicated that the groups may be plant-specific or were lost in yeasts. Interestingly, the subfamilies UBC4/5 or UBC3/7 shared two highly identical paralogous yeast genes, respectively. For example, during the phylogenetic analysis of CpUBC genes using MEGA 5.0, UBC4 and UBC5 always clustered into the same subgroup, as did UBC3 and UBC7. According to previous reports [26, 27, 47], we designated the two clades as subfamilies UBC4/5 and UBC3/7.

### Organ- and ripening stage-specific expression of CpUBC genes

**Fig 4:**
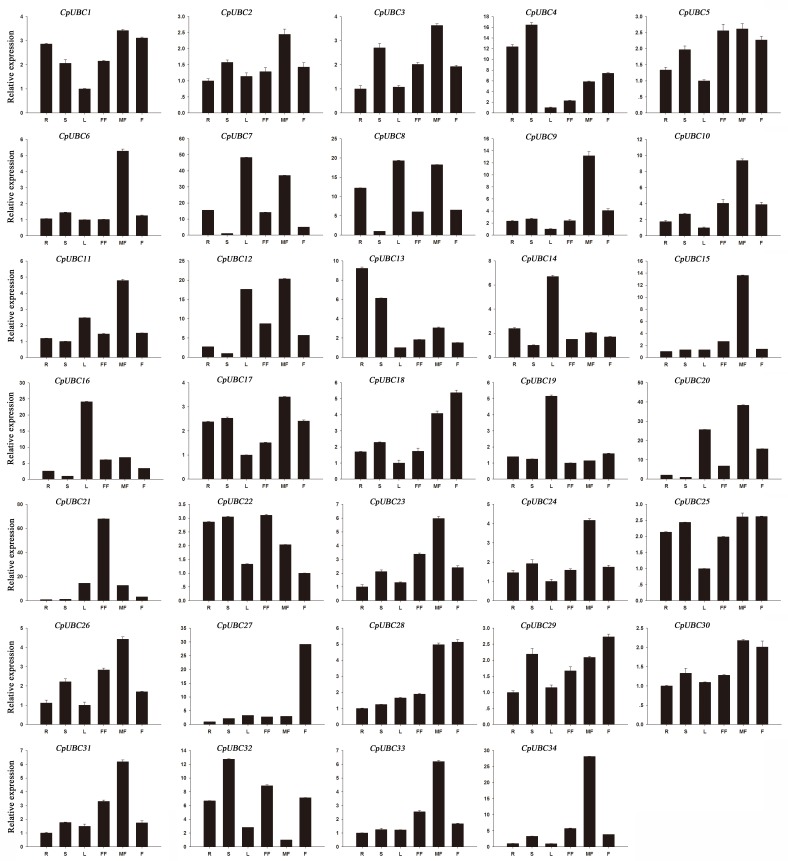
Tissue-specific expression of selected CpUBC genes. The x-axis indicates time course/treatment, and the y-axis indicates the relative expression level. Error bars indicate standard deviations of independent biological replicates (n ≥ 3). R, root; St, stem; L, leaf; T, tassel, Ys, young seed; and Si, silk.

All 34 of the candidate CpUBC genes were differentially expressed among the six papaya organs (Fig 4). In addition, we found that the expression of 13 (*CpUBC1*/*4*/*5*/*6*/*9*/*10*/*13/17*/*18*/*24*/*25*/*26*/*34*), 11 (*CpUBC2*/*3*/*15*/*21*/*23*/*27*/*28*/*29*/*30*/*31*/*33*), and seven (*CpUBC7*/*8*/*11*/*12*/*14*/*16*/*20*) genes were lowest in leaves, roots, and stems, respectively, and that the expression of only one gene was lowest in the male flowers (*CpUBC32*) and female flowers (*CpUBC19*), respectively. Meanwhile, 19 (*CpUBC1*/*2*/*3*/*5*/ *6*/*9*/*10*/*11*/*12*/*15*/*17*/*20*/*23*/*24*/*26*/*30*/*31*/*33*/*34*) and two genes (*CpUBC21* and *CpUBC22*) were highly expressed in male flowers and female flowers, respectively, which implicated their involvement in the development of floral sex organs. Four (*CpUBC25*/*27*/*28*/*29*), five (*CpUBC7*/*8*/*14*/*16*/*19*), and two (*CpUBC4* and *CpUBC32*) genes were highly expressed in fruits, leaves, and stems, respectively, and a single gene (*CpUBC13*) was highly expressed in roots. Therefore, our results suggested that CpUBC genes play multiple roles in papaya development.

**Fig 5:**
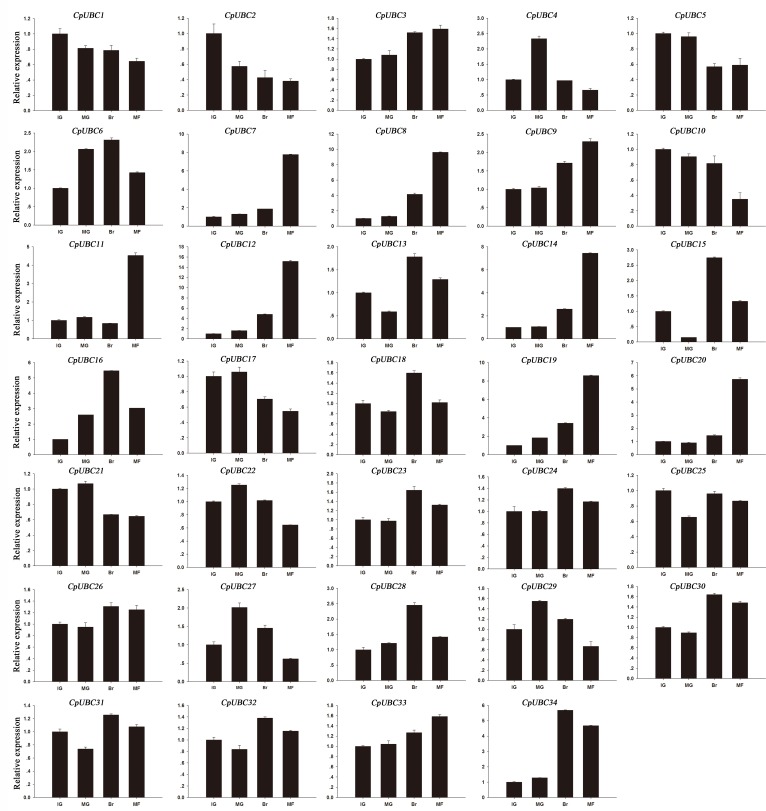
Development stage-specific expression of selected CpUBC genes. The x-axis indicates time course/treatment, and the y-axis indicates the relative expression level. Error bars indicate standard deviations of independent biological replicates (n ≥ 3). IG, immature green; MG, mature green; Br, breaker; and MF, mature fruit.

In the present study, we also analyzed the expression of the 34 CpUBC genes at different fruit ripening stages. We found that 13 (*CpUBC4/6/7/8/9/11/12/14/16/19/20/28/34*) and two (*CpUBC2* and *CpUBC10*) of the genes showed higher or lower expression during the progress of papaya fruit ripening, respectively, whereas the expression of the remaining genes was not significantly different (Fig 5). Of the 13 genes that were higher expressed in the fruit, the expression levels of eight genes (*CpUBC7/8/9/11/12/14/19/20*) were slightly increased at the IG, MG, and Br stages and most highly expressed at the MF stage. Meanwhile, the expression of four genes (*CpUBC6/16/28/34*) continuously increased during the first three stages of fruit ripening, before being decreased at the MF stage, and the expression of *CpUBC4* was increased at MG stage and then reduced at the two late stages. These results suggest that the papaya E2s maybe playing a significant role in fruit development and ripening.

## Discussion

Ripening affects the quality and nutritional contents of fleshy fruits and is a crucial process in the development of climacteric fruits [48]. In recent years, the study of genes involved in fruit ripening has rapidly progressed; however, most research has been conducted in tomato. In addition, E2 proteins are critically important in many aspects of plant growth and development; however, considering the potential functional significance of E2 proteins, only a few E2 family members have been described in higher plants[27, 31, 38], and only a few studies have investigated the role of the UBC gene family in fruit ripening [29, 31].

In the present study, we identified genes encoding 34 *C*. *papaya* UBC (CpUBC) proteins (Table 1), which includes two RUB-conjugating enzymes (CpUBC18 and CpUBC29), one SUMO-conjugating enzyme (CpUBC11), and six UEV proteins (CpUBC2/14/16/19/24/25). Similar results have also been reported by other studies. For example, 16 UBC proteins were identified in *S*. *cerevisiae*, including one RUB-conjugating enzyme (ScUBC9), one SUMO-conjugating enzyme (ScUBC12), and two UEV protein (ScMMS2, NP_011428 and ScSTP22, NP_009919) [49]. In addition, a total of 48 UBC domain-containing proteins have been identified in *Arabidopsis*, of which two are RUB-conjugating enzymes (RCE1, At4g36800 and RCE2, At2g18600), one is a SUMO-conjugating enzyme (AtSCE1, At3g57870), and eight others are UEVs, thus leaving 37 potential E2s [27]. Among 48 rice E2s, three proteins (OsUBC1/2/3) were identified as RUB E2s, three SUMO E2s (OsUBC4/5/6), and four UEVs (OsUBC28/29/30/31) [28]. Meanwhile, of the 52 SlUBC proteins in tomato, there are three SUMO E2s (SlUBC8/23/51) and five RUB E2s (SlUBC10/13/18/29/50) [31]; and of the ZmUBC proteins in maize, there are three RUB E2s (ZmUBC-37/40/43), eight SUMO E2s (ZmUBC-01/04/11/12/51/69/73/75), and six UEVs (ZmUBC-08/04/31/34/53/74) [30]. The number of CpUBC genes is far less than the 48, 48, 52, 75, and 74 UBC genes reported from the *Arabidopsis* [27], rice [28], tomato [31], maize [30], and banana genomes [29], respectively. The genome sizes of *Arabidopsis*, rice, tomato, maize, and banana are ~125, ~466, ~900, ~2300, and 523 Mb, respectively. However, although the genome sizes of rice and tomato are 3.7 and 7.2 times larger than that of *Arabidopsis*, respectively, the genomes of the three species contain a similar number of UBC genes (45, 48, and 52, respectively); and although the genome size of maize is 4.3 times larger than that of banana, the genomes of these two species also contain a similar number of UBC genes (75 and 74, respectively). Therefore, the difference in number of UBC genes among these species cannot be explained by genome size.

Alternatively, gene duplication events have been demonstrated to play a significant role in the expansion of gene family members in genomes [38], and an increasing number of studies has shown that segmental duplications are largely responsible for the expansion of gene families in maize, such as the CCCH, HD-Zip HSF, bZIP, and PRX gene families[50–53]. Research has also shown that segmental duplications are the main contributor to the expansion of UBC genes in maize and banana [29, 30]. However, owing to a lack of information regarding the chromosomes of papaya, we were unable to perform segmental and tandem duplication analysis in papaya.

Through systematic phylogenetic analyses, the present study provides a detailed classification and nomenclature of papaya E2s. The phylogenetic analysis categorized the 34 CpUBCs into 17 discrete groups, which was similar to the number of groups described in other species, such as tomato, maize, and banana [29–31]. However, there were still some minute differences between the topologies of the UBC genes in papaya and the other species. For example, there are 15 and 13 UBC groups in tomato and banana, respectively, not including the UEV subgroups [29, 31]. However, in *Arabidopsis*, rice, and maize, the UEV subgroups were taken into account [27]. In our study, to better understand the papaya E2s, the CpE2s were divided into 17 groups, which included three independent UEV groups. The corresponding homologs of AtUBC21 and AtUBC22 were grouped together in maize [30], banana [29], and papaya, whereas the corresponding homologs (and orthologs) were separated into two groups in tomato [31]. Additionally, homologs of AtUBC31, grouped together in the UBC4/5 group in maize and banana [29, 30], were not grouped in any groups in our study. These differences could have resulted from different parameter settings during the phylogenetic analyses.

In addition, the number of UBC genes was obviously different among the groups. The largest groups (UBC4/5) included eight members, whereas the UBC1, UBC2, UBC6, UBC9, UBC10, UBC13, UBC14, and UBC15 groups were only represented by one member each. The UBC4/5 groups was also the largest in other species, which indicates that, in plants, the group may possess more diverse functions than other groups. Phylogenetic data also suggested that the UBC9 and UBC12 groups were expanded in monocots but not in *Arabidopsis*, and previous research has shown that the genomes of maize and rice each contain eight and three members of the UBC9 and UBC12 groups, respectively [28, 30]. However, in papaya, we only identified one and two members in these groups. Furthermore, we also identified three UBC groups (UBC14, UBC15, and UBC16) in *Arabidopsis* and papaya for which no probable homologs exist in budding yeast; however, all three groups have potential homologs in animals [54], which indicates a possible gene loss during yeast evolution.

Although the genome sequence of papaya has been reported, the identification and functional studies of papaya genes have proceeded at a slow pace. Analysis of the temporal and spatial expression patterns of genes may provide useful information for characterizing their functions. Previous studies have shown that UBC genes have different expression patterns in different organs. In *Arabidopsis*, for example, *AtUBC1* and *AtUBC2* are ubiquitously expressed in roots, leaves, flowers, and seedlings, and the double mutant *atubc1-1atubc2-1* exhibits a dramatically reduced number of rosette leaves and an early-flowering phenotype [55]. In banana, *MaUBC10/11/33/34/61* are highly expressed in most organs, especially in roots, stems, leaves, and *MaUBC6/11/34/35/45/61* were highly expressed in stems. In addition, 12 *MaUBC* genes (*MaUBC13/18/29/33/34/36/43/46/48/53/67/70*) were predominantly expressed in roots [29]. Meanwhile, in the present study, we found that 19 and two genes were highly expressed in male and female flowers, respectively, which suggests that the genes may be involved in the development of floral sex organs, and various other CpUBC genes were specifically higher expressed in other tissues, which that CpUBC genes play multiple roles in the development of papaya.

Previous studies have demonstrated that ubiquitin-conjugating enzymes (E2s) are critically important in many aspects of plant growth and development, as well as in physiological processes, such as stress responses [56]. For example, *OgUBC1* from wild rice is involved in cellular responses to biotic and abiotic stresses [56], and three (*OsUBC2/5/18*) and five UBC genes (*AtUBC13/17/20/26/31*) from rice and *Arabidopsis*, respectively, are significantly reduced in response to salt and drought stress, whereas only three rice genes (*OsUBC13/15/45*) are significantly upregulated [38]. Although the function of E2s in plant development and stress responses have been well clarified, reports about the function of protein ubiquitination in fruit ripening remains extremely rare, except in tomato and banana [29, 31]. In tomato, a total of six UBC genes (*SlUBC6/8/24/32/41/42*) were directly regulated by the fruit-ripening regulator RIN, which suggests that specific UBC genes might be involved in fruit ripening, and in banana, five UBC genes (*MaUBC1/9/70/68/71*) exhibited approximately 10- to 40-fold higher levels of expression at the fifth stage than at other stages of fruit ripening, whereas seven other UBC genes (*MaUBC8/16/17/33/34/56/61*) exhibited continuously increasing expression throughout the fruit development. In the present study, 13 (*CpUBC4/6/7/8/9/11/12/14/16/19/20/28/34*) and two (*CpUBC2* and *CpUBC10*) of the 34 CpUBC genes were up- or downregulated during the progression of papaya fruit ripening, respectively (Fig 5), and our results indicated that the expression patterns of some of the CpUBC genes, such as *CpUBC17* and *CpUBC56*, was related to their phylogenetic relationships. By contrast, the expression patterns of some paralogs, such as *CpUBC8* and *CpUBC23*, were quite different. Meanwhile, we also found that the genes with close phylogenetic relationships that exhibited similar expression patterns in banana, tomato, and papaya. For example, our data indicated that the *CpUBC23* orthologs *MaUBC3/8* and *SlUBC32* were similarly expressed during fruit ripening. However, *CpUBC23* was not considered differentially expressed genes in the present study since its expression was upregulated by less than two-fold. *MaUBC1*, *MaUBC9*, *SlUBC41*, *SlUBC42*, and *CpUBC34*, which belong to the *UBC8* subgroup, also exhibited similar expression patterns during fruit ripening. Therefore, taken together, our results suggest that the papaya E2 family genes might be participated in the regulation of fruit development and ripening processes in papaya.

## Conclusions

In the present study, we describe the genome-wide identification and analysis of UBC genes in papaya. A total of 34 putative CpUBC genes were identified, and phylogenetic analysis indicated that genes could be divided into 17 subfamilies. Analysis of exon-intron junctions and sequence motifs revealed high levels of conservation within and between phylogenetic groups. In addition, all the CpUBC genes were detected in roots, stems, leaves, male and female flowers, and mature fruit, although some genes showed higher or lower expression in one or several specific organs. Similar to the results in tomato and banana, we also found that the expression of 13 and two CpUBC genes were incresesd or decreased during one and two ripening stages, respectively. Therefore, the results of the present study suggest that CpUBC genes are involved in the regulation of fruit development and ripening processes. The results also provide novel insights into the function of plant UBC genes and will facilitate further investigation of the roles of the UBC genes in fruit ripening.

## Supporting information

S1 Table Quantitative RT-PCR primers. (DOC)
