# Supplementary material for: Characterization and expression analysis of genes encoding ubiquitin conjugating domain-containing enzymes in Carica papaya
Source: PLoS One. 2017 Feb 23;12(2):e0171357. doi: 10.1371/journal.pone.0171357 (PMC5322903; doi:10.1371/journal.pone.0171357)
Supplement: S1 Table — (DOC) [file pone.0171357.s001.doc]

**S1 Table. Quantitative RT-PCR primers**

| **Genes** | **Primers used in Semi-quantitative RT-PCR(5′→3′)** | |
| --- | --- | --- |
| *CpUBC1* | Forwards： | TCGACTCAAGCCAGTCTCCT |
|  | Reverse： | CATTGAAGAATCCCCCTTCA |
| *CpUBC2* | Forwards： | TTGGAAGAGCTTGAGAGGGG |
|  | Reverse： | CTTCAACTGGTAGATGCGCC |
| *CpUBC3* | Forwards： | CCATTTAAGCCTCCCAAGGT |
|  | Reverse： | TGAGCAATTTCTGGCACAAG |
| *CpUBC4* | Forwards： | TTAAGCCTCCCAAGGTAGCA |
|  | Reverse： | AGGATCCGTCAACAATGAGC |
| *CpUBC5* | Forwards： | TCAAAGTCACCCCAAAGTCC |
|  | Reverse： | TGATATTGGGGTGCCAAACT |
| *CpUBC6* | Forwards： | TATCCTATGGAAGCCCCACA |
|  | Reverse： | TTGTCTTCAGGGCGTTTCTT |
| *CpUBC7* | Forwards： | GGACCAAGTGACAGCCCTTA |
|  | Reverse： | AGGACTCCACTGCTCCTTGA |
| *CpUBC8* | Forwards： | TACCGCCCAGAAACTTTGAC |
|  | Reverse： | CCCTAGCAACCCTTTGAACA |
| *CpUBC9* | Forwards： | GAAGGAGCAATGGAGTCCTG |
|  | Reverse： | AGGATCGAGCGGTAGTCTCA |
| *CpUBC10* | Forwards： | CGATTTGTTTCCCGAATGTT |
|  | Reverse： | CTTCTGAATTTGCCGGAGAG |
| *CpUBC11* | Forwards： | CCAGCCATTACTGTGAAGCA |
|  | Reverse： | GGATATTGTTTGGCCTGCTG |
| *CpUBC12* | Forwards： | CGGACAGCTCCAAAACAAAT |
|  | Reverse： | TGCCTGTTTCTGTGATGAGC |
| *CpUBC13* | Forwards： | TACAAGGGTGCCAAGAATCC |
|  | Reverse： | GACTGCCCGTAAAAGATCCA |
| *CpUBC14* | Forwards： | ACCCTATTCACCCTCCTTGC |
|  | Reverse： | GAGGAAGGAGTCTGGAGTCG |
| *CpUBC15* | Forwards： | AAGGAGTTGCAGGGAGATCC |
|  | Reverse： | TCATCTGGGTTTGGATCGGT |
| *CpUBC16* | Forwards： | AGGCGGAGGAGGAAATTGAA |
|  | Reverse： | AGCCTCAAAAGCATCCTCCT |
| *CpUBC17* | Forwards： | TGTCTCTCATGATGGGTGGA |
|  | Reverse： | TGGAAGCACATGGTCTCAAA |
| *CpUBC18* | Forwards： | CCAAATGGCAAGGATGACTT |
|  | Reverse： | GGCAGACATTTCCTTCCAAA |
| *CpUBC19* | Forwards： | TTGTGAATATGGCGCATGGG |
|  | Reverse： | GGTTTTGCCCTCGTTTTCCT |
| *CpUBC20* | Forwards： | TTGGGGACATGAGAGAGCTT |
|  | Reverse： | TGAATCCCACCTTGGACAAT |
| *CpUBC21* | Forwards： | GCTTCCATCCCAATGTTGAT |
|  | Reverse： | AGTTGGGCTGCTTGAGTGTT |
| *CpUBC22* | Forwards： | CTGGCAAGATGTTGCTTGAA |
|  | Reverse： | GGAGGGGCTAACACAGATGA |
| *CpUBC23* | Forwards： | CTTGGAAGGATATGCCTGGA |
|  | Reverse： | CCTCTGCCTCATTCGTCTTC |
| *CpUBC24* | Forwards： | AGCCACCAAGTGTTCGATTC |
|  | Reverse： | TCCATCGTGTACTCTCGCTG |
| *CpUBC25* | Forwards： | GGAAAGGAGCTTCCTCGTCT |
|  | Reverse： | GCCACCTTCATACGGAGTTC |
| *CpUBC26* | Forwards： | TTATGGGTCCTCCTGACAGC |
|  | Reverse： | ATGTCCAGGCAAATGCTACC |
| *CpUBC27* | Forwards： | TCCATGGACCAAAAGAAAGC |
|  | Reverse： | GGCCTTTCCGTTCCAAGTAT |
| *CpUBC28* | Forwards： | ATCATCATCGGCTCAGCTCA |
|  | Reverse： | CTGAAAACACCACCTTCCCA |
| *CpUBC29* | Forwards： | GCGGGAAGATTGGAAACCTG |
|  | Reverse： | CCACATACCCACCAGCCATA |
| *CpUBC30* | Forwards： | CACTCCTTGGGATGGAGGTA |
|  | Reverse： | AAGAATAGCCGCAACATCGT |
| *CpUBC31* | Forwards： | CCGCCAACTGTCCATTTTGT |
|  | Reverse： | GATTCGGGTCACAGAGCAAG |
| *CpUBC32* | Forwards： | CTCCCAACTATCCGAACAGC |
|  | Reverse： | TTTGTATGGACAGGCATCCA |
| *CpUBC33* | Forwards： | ATCCCAATCCATCAGATCCA |
|  | Reverse： | GCAATTGCCTCATCATCAGA |
| *CpUBC34* | Forwards： | TGGACCCAGTGAGAGTCCTT |
|  | Reverse： | GGGCTCCATGTCTGATTGAT |
| *CpActin* | Forwards： | TCACTACGACTGCCGAGCGAG |
|  | Reverse： | GAGCCACCACTGAGGACAACATTAC |
